# Rupture of an epidural filter connector during bolus administration of local anesthetic: a case report

**DOI:** 10.1186/s12871-021-01372-z

**Published:** 2021-05-12

**Authors:** Daniel A. Nahrwold, Aaron R. Muncey, Nasrin N. Aldawoodi, Raymond M. Evans, Jamie P. Hoffman

**Affiliations:** grid.170693.a 0000 0001 2353 285X H. Lee Moffitt Cancer Center & Research Institute, University of South Florida Morsani College of Medicine, 12902 USF Magnolia Dr, Tampa, FL 33612 USA

**Keywords:** Epidural catheter, Filter connector, Equipment, Local anesthetic, Pressure, Safety, Injury, Infection

## Abstract

**Background:**

Epidural catheters are routinely placed for many surgical procedures and to treat various pain conditions. Known complications arising from epidural catheter equipment malfunction include epidural pump failure, epidural catheter shearing, epidural catheter connector failure, epidural filter connector cracking, and loss-of-resistance syringe malfunction. Practitioners need to be aware of these potentially dangerous complications and take measures to mitigate the chances of causing significant patient harm. We report on the complete breakage of an epidural filter connector during epidural bolus administration of local anesthetic by hand with a syringe.

**Case presentation:**

A B. Braun Perifix® epidural catheter was placed in a 73-year-old male scheduled for radical prostatectomy. During the operation, a continuous infusion of local anesthetic was administered through the epidural catheter in addition to general endotracheal anesthesia. At the conclusion of surgery and after extubation, the patient endorsed incisional pain. The epidural filter connector broke in half as a bolus of local anesthetic was administered by hand with a syringe. The local anesthetic sprayed widely throughout the room as the fragmented epidural filter connector became a projectile object that recoiled and struck the patient.

**Conclusions:**

This incident placed the patient and surrounding healthcare providers at substantial risk for injury and infection from the fractured epidural filter connector becoming a projectile object and from the local anesthetic spray. The most plausible cause of this event was from a large amount of pressure being applied to the filter connector. This may have occurred by excessive force being applied by hand to the syringe, by the presence of a clogged filter, or by the catheter being kinked or blocked proximal to the filter. Being aware of this deleterious complication and potentially modifying existing epidural bolus techniques, such as using smaller syringes with less applied force and checking all epidural components vigilantly prior to and during bolus administration, can help anesthesia providers deliver the safest possible care to patients with epidural catheters.

## Background

Epidural catheters are commonly placed for a wide range of surgical procedures and to aid in the management of acute and chronic pain. Complications related to epidural catheter placement such as epidural hematoma, nerve injury, and infection are well known and described in the literature [1]. Complications related to epidural equipment problems or defects are lesser known and limited to case reports in the literature describing epidural pump failure, catheter connector failure, filter connector cracking, epidural catheter shearing and breakage, and loss-of-resistance syringe malfunction [2–7].

Epidural boluses of local anesthetic or opioid are routinely administered to patients in order to enhance pain control. This may be accomplished through the epidural catheter connector or filter connector using the bolus feature of an infusion pump or by hand using a syringe. Epidural filter connectors have traditionally been used to decrease bacterial intrusion into the epidural space and to block debris such as glass or plastic from entering [8]. We describe the first case to ever be reported in the literature where the epidural filter connector split in two while a bolus of local anesthetic was being administered to the patient by hand.

Written HIPAA consent for the publication of this case report was obtained from the patient. This manuscript adheres to the applicable EQUATOR guidelines.

## Case Presentation

A 73-year-old male long-term cigarette smoker with no significant past medical history presented for right nephroureterectomy and radical cystoprostatectomy with ileal conduit diversion for bladder and ureteral cancer. Prior to the induction of general anesthesia, a T10-11 epidural catheter was placed to be utilized intraoperatively and for post-operative pain management.

The epidural catheter was placed with components from a B. Braun Perifix® continuous epidural anesthesia tray (B. Braun Medical Inc., Bethlehem, Pennsylvania, USA). A 20-guage closed tip epidural catheter was inserted into the epidural space at T10-11. This was accomplished using a loss-of-resistance technique through a midline approach with a 17-gauge Tuohy needle and a Perifix™ plastic luer slip loss-of-resistance syringe. The epidural catheter was inserted five cm into the epidural space. The epidural catheter was then connected to a clamp style catheter connector which was then attached to a 0.2 μm filter connector.

A test dose of 3mL of 1.5% lidocaine with 1:200,000 epinephrine was administered without significant resistance through the filter connector with a 20mL luer lock plastic syringe. The test dose was negative, general anesthesia was induced, and the procedure began as planned. The anesthetic was maintained in this patient using a combination of inhaled sevoflurane through an endotracheal tube and a continuous infusion of 0.0625% bupivacaine through the epidural catheter. A B. Braun Perfusor® Space Infusion Pump was used to deliver the continuous epidural infusion. Infusion rates varied between 2 and 8mL per hour for the duration of the operation, and high-pressure alarms on the pump never activated during the case.

**Fig. 1:**
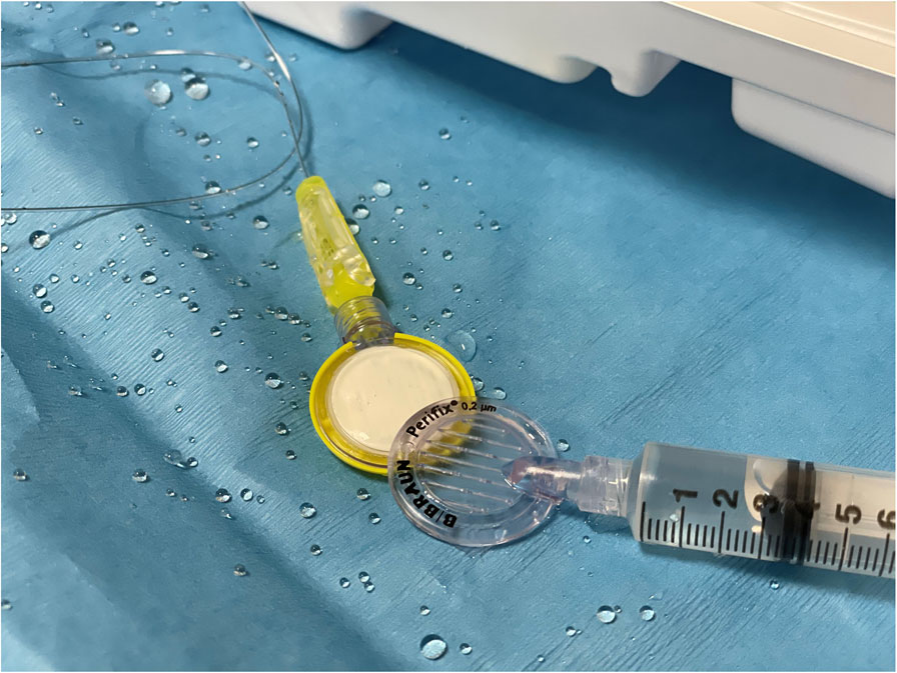
A staged image showing the top of the epidural filter connector separated from the base with surrounding local anesthetic spray

At the conclusion of an uneventful and successful surgical procedure, the patient was extubated smoothly. The patient endorsed incisional pain, and the patient’s blood pressure, heart rate, and respiratory rate were all mildly elevated from baseline. The continuous epidural infusion was stopped. A bolus of 5mL of 0.25% bupivacaine with 1:200,000 epinephrine was delivered by hand using a BD 10mL Luer-Lok™ syringe (Becton, Dickinson and Company, Franklin Lakes, New Jersey, USA) via the epidural filter connector. Midway through the hand delivered bolus, the epidural filter connector top popped off from the rest of the mechanism spraying the contents of the syringe several feet in multiple directions (Fig. 1). The bottom half of the epidural filter connector that remained connected to the clamp style catheter connector and epidural catheter itself recoiled and struck the patient near his shoulder which did not cause any immediate noticeable harm.

It was estimated that the patient received 2mL of the epidural bolus before the filter connector broke. An additional 3mL was then administered to the patient via the clamp style catheter connector without significant resistance. The patient was then taken to the post-anesthesia care unit (PACU) where a new filter connector was attached to the clamp style catheter connector. A BD Alaris™ patient-controlled analgesia (PCA) pump was then used to deliver 0.0625% bupivacaine with 10mcg/mL hydromorphone at 6mL/hour with a 2mL every 20 minutes demand option. The epidural catheter was removed on post-operative day (POD) 3 as the patient transitioned to oral pain medications. The patient was discharged from the hospital on POD 5 in stable condition.

## Discussion

Epidural catheter and related equipment design and innovation has steadily progressed over many decades [9]. Therefore, complications related to epidural equipment disturbances are uncommon and limited to case reports describing epidural pump failure, catheter connector failure, filter connector cracking, epidural catheter shearing and breakage, and loss-of-resistance syringe malfunction [2–7]. To our knowledge, this is the first description in the literature of an epidural filter connector breaking in half during bolus administration of local anesthetic by hand with a syringe.

Total volume of local anesthetic and increasing patient age allow for a greater distribution and spread of sensory blockade after epidural injection, while speed of delivery and pressure exerted during local anesthetic injection through an epidural catheter have lesser effects [10]. Though it is not our aim or practice, it is possible that too much pressure was exerted on the filter connector as the syringe plunger was depressed by hand during bolus administration. Additionally, syringe size is inversely proportional to the amount of pressure that can be generated when injecting into tissues and cavities such as the epidural space [11]. In our case, we used a 10mL syringe for injection which would generate less pressure than 3mL and 5mL syringes that are also commonly used to dose epidural catheters. It may be prudent to use pressure monitoring devices such as the B-Smart™ in-line manometer (B. Braun Medical Inc., Bethlehem, Pennsylvania, USA) or CompuFlo® computerized injection pump technology (Milestone Scientific Inc., Livingston, New Jersey, USA) to avoid high injection pressures which could lead to both tissue and nerve injury as well as disrupt the integrity of the epidural catheter equipment as we experienced in our case [12].

Epidural filter connectors are widely used when placing epidural catheters in order to minimize the risk of bacterial contamination of the epidural space [8]. They are also placed to mitigate the risk of particulate debris such as glass or plastic from syringes, vials, or other epidural equipment from entering the epidural space. Particulate matter entering the epidural space leading to nerve impingement or injury could happen easier without a filter connector in place but has also happened with a filter connector attached [13]. There are no case reports of this happening with modern epidural filters. In our case, it is possible that some debris entered and clogged the filter leading to increased injection pressures and subsequent rupturing of the filter connector. The filter membrane of the connector appeared intact after the apparatus broke though we cannot be certain that some degree of clogging took place on a microscopic level.

It is also possible that the filter connector had a manufacturer’s defect which led to a weakening or blocking of the apparatus. This seems to be a rare occurrence, and a thorough review of the literature revealed a single letter correspondence describing a redundant and misaligned filter membrane causing a blocked epidural filter connector [14]. We reached out to B. Braun Medical Inc., and the company had never heard of such an event and also stated that their equipment adheres to the highest standards of manufacturing and quality control checks.

The epidural catheter and clamp style connector which are proximal to the filter connector could have caused obstruction leading to increased pressure within the system and eventual filter connector breakage. It is unlikely that the clamp style connector was damaged because after the filter connector broke, injection of local anesthetic through the clamp style connector proved to be easy and without significant resistance. Epidural catheters themselves can experience coiling, curling, kinking, knotting, and stretching [15, 16]. They can also develop blood clots at the tip [17]. Again, these would be unlikely causes for filter connector rupture in our case, as injection through the clamp style connector was smooth. Additionally, when the epidural catheter was removed on POD 3, it was found to be undamaged and completely intact. Another potential cause of proximal obstruction within the components of the epidural system is counterpressure build-up where pressure builds within the epidural space and injecting becomes more difficult as increased amounts fluid such as local anesthetic or saline are injected [18]. This would occur rarely within the actual epidural space and would be more likely if the epidural catheter was inserted into another tissue plane or muscle. Again, our injection was smooth once the epidural filter connector was removed making counterpressure build-up a less likely reason for our filter rupture.

Another concerning aspect of the epidural filter connector breaking is that the top popped off from the rest of the filter causing bupivacaine to be sprayed in a wide path. At the time, only two practitioners were near the patient and both were wearing facemasks and eye protection. The bottom half of the filter connector remained connected to the clamp style catheter connector and thus to the epidural catheter itself as it broke away from the top half of the filter connector. No one got injured as the two parts disconnected though the patient and the two providers nearby did get mildly saturated with the bupivacaine spray. The providers’ eye protection limited any fluid from entering their eyes, and the patient’s eyes were closed at the time. The risk of harm from bupivacaine entering a person’s eyes is relatively low but can be damaging in those with pre-existing eye conditions or if the fluid is sprayed with high force and in large quantities [19]. Additionally, there was a risk of introducing infection to the providers in the room and the patient with the now compromised connector and from the local anesthetic spray.

Our incident seems most likely related to enough pressure being generated by hand to disrupt the integrity of the filter connector. It is very unlikely that an epidural infusion pump itself could generate enough force to damage a filter connector, and modern pumps stop infusing when pressure limits are reached. Also, this case raises the question of whether providers should be bolusing epidural catheters by hand through the clamp connector rather than through the filter connector. A review of the literature does not aid in making this determination. There is reassurance in knowing that bolusing epidurals by hand with syringes is not a common reason for clamp connector breakage or disconnect [20].

Epidural catheter equipment malfunction is uncommon but has the potential for serious consequences when it does occur. Bolusing an epidural catheter with a syringe by hand could generate enough pressure to disrupt the integrity of the filter connector. A broken filter connector becoming a projectile object as well as spray of local anesthetic could harm the patient and surrounding personnel. Careful consideration should be taken in determining how much force to use when bolusing an epidural catheter with a syringe by hand and whether to administer the local anesthetic through the filter connector itself or through the other connector that attaches directly to the epidural catheter.

## Data Availability

Not applicable.

## Abbreviations

PACU: Post-anesthesia care unit
PCA: Patient-controlled analgesia
POD: Post-operative day
